# Foot problems in people with gout in primary care: baseline findings from a prospective cohort study

**DOI:** 10.1186/s13047-015-0090-9

**Published:** 2015-07-23

**Authors:** Edward Roddy, Sara Muller, Keith Rome, Priyanka Chandratre, Samantha L. Hider, Jane Richardson, Milisa Blagojevic-Bucknall, Christian D. Mallen

**Affiliations:** Research Institute for Primary Care and Health Sciences, Keele University, Keele, Staffordshire ST5 5BG UK; Division of Rehabilitation & Occupation Studies, Health & Rehabilitation Research Institute, AUT University, Auckland, 1020 New Zealand

**Keywords:** Foot, Gout, Pain, Hallux valgus, Epidemiology

## Abstract

**Background:**

Foot problems are common in people with gout yet the prevalence of current foot problems in people with gout and the burden they present to healthcare systems is not known. This cross-sectional study aimed to determine the prevalence and associations of hallux valgus, foot pain and disability in people with gout, and to assess the frequency with which foot problems lead to consultation with healthcare professionals.

**Methods:**

Adults registered with 20 general practices who had consulted their GP about gout or been prescribed allopurinol or colchicine in the preceding two years were mailed a questionnaire. Prevalence of hallux valgus, foot pain in the last month, and disabling foot pain in the mailed population were ascertained using validated instruments and estimated by inverse-weighted logistic regression. Associations with socio-demographic, comorbid and gout-specific factors were examined using logistic regression. Participants were asked if they had seen health care professionals for foot problems within the preceding 12 months.

**Results:**

One thousand one hundred eighty-four questionnaires were received (response 66 %). Prevalence of hallux valgus was 36.3 %, foot pain in the last month 22.3 % and disabling foot pain 14.5 %. Hallux valgus associated with age (adjusted OR 1.47 per 10-year increase, 95 % CI 1.26, 1.72) and female gender (2.03; 1.31, 3.15). Foot pain in the last month associated with age (1.24; 1.00, 1.55), obesity (BMI 30.0–34.9 2.67; 1.32, 5.38; BMI ≥ 35.0 3.16; 1.44, 6.93), mild depression (2.04; 1.09, 3.81) and polyarticular gout attacks (1.86; 1.18, 2.95). Disabling foot pain associated with age (1.42; 1.08, 1.87), obesity (BMI 30.0–34.9 3.73; 1.54, 9.09; BMI ≥ 35.0 4.36; 1.64, 11.64), depression (mild 2.63; 1.25, 5.53; moderate 3.53; 1.11, 11.26) and ischaemic heart disease (2.45; 1.32, 4.53). In the previous 12 months, 495 (42.8 %) reported consulting their GP about their feet and 281 (23.7 %) a podiatrist/chiropodist.

**Conclusions:**

Foot problems are common in people with gout and frequently lead to healthcare consultation. Hallux valgus has similar associations to those seen in the general population, whereas foot pain associates with obesity and gout characteristics, and disabling foot pain with obesity and comorbidity. Patient assessment should consider foot problems and offer specific treatment where relevant.

## Background

Gout is the most prevalent inflammatory arthropathy and affects 2.5 % of adults in the UK [1]. It shows a striking tendency to affect the joints of the foot and ankle complex with involvement of the 1^st^ metatarsophalangeal joint (MTPJ) being the clinical hallmark of gout. As many as 89 % of patients experience involvement of the 1^st^ MTPJ at some point in the disease course, with 56–78 % of first attacks affecting this joint [2]. The midfoot joints and ankle are the next most frequent sites of acute attacks. However, whilst acute attacks of excruciating joint pain and swelling are characteristic of crystal synovitis, gout also has well-recognised chronic manifestations including an erosive, destructive arthropathy and a pathogenetic link with osteoarthritis (OA) [3]. Both hallux valgus deformity and chronic big toe pain are more common in people with gout than in age- and gender-matched control subjects [4]. A recent small hospital-based study found that people with chronic gout have greater levels of general and foot-specific disability, pain and impairment, slower walking velocity, reduced step and stride length, reduced peak plantar pressure under the hallux, and higher mid-foot pressure-time integrals [5].

Although chronic foot problems appear to be more common in people with gout than in people of a similar age and gender without gout, the reasons why people with gout experience these problems are less clear. Age, gender, obesity, footwear, foot posture, joint pain and OA have been suggested to be risk factors for hallux valgus and foot pain in the general population [6–15]. Footwear worn by people with gout frequently provides poor cushioning, lack of support, lack of stability, and motion control, and demonstrates excessive wear patterns [16]. However, it is not known whether co-morbid disease, gout-related characteristics and indicators of gout severity are risk factors for chronic foot problems in gout.

Whilst it is well-recognised that foot involvement is a cardinal feature of gout and the vast majority of gout sufferers experience acute attacks affecting the foot [2], there are few studies of the prevalence of current foot problems in people with gout and the frequency with which these lead to consultation with healthcare professionals is not known. However, in general population studies not specific to gout, 12 % of people with foot problems consulted their GP about them over an 18-month period and 18 % consulted a podiatrist over 12 months [17, 18], suggesting that foot problems present a significant burden to healthcare systems. Such information is needed to inform healthcare provision.

The objectives of this study were (i) to determine the prevalence of hallux valgus, foot pain and disabling foot pain amongst people with gout, (ii) to describe the anatomical location of foot pain, (iii) to investigate which demographic, lifestyle, psychological, co-morbid and gout-related factors are associated cross-sectionally with foot problems in gout, and (iv) to describe the 12-month prevalence of consultation with a general practitioner, physiotherapist or podiatrist/chiropodist for foot problems.

## Methods

### Study design

This paper uses baseline data from a 3-year primary care-based prospective observational cohort study [19]. Ethical approval was obtained from North West - Liverpool East Research Ethics Committee (REC reference number: 12/NW/0297).

### Study population

Gout patients aged 18 years and older were recruited from 20 general practices across the West Midlands, UK. Electronic primary care records in the participating practices were searched over the preceding two years to identify patients with a Read code for a consultation for gout or a prescription of allopurinol or colchicine. Read codes are a coded hierarchy of clinical codes, based on ICD-9 codes, and are widely used in primary care in the UK.

### Data collection

Eligible patients were mailed a self-complete questionnaire that requested information about aspects of gout, foot problems and general health. Specific questions about gout included age at gout diagnosis, whether an attack of gout was being experienced at the time of questionnaire completion, number of attacks experienced in the preceding 12 months, history of gout attacks affecting more than one joint (oligo/polyarticular gout) and current use of allopurinol. A validated line-drawing instrument was used to assess self-reported hallux valgus [20]. This instrument consists of five drawings for each foot, depicting a sequential increase in the hallux valgus angle of 15° and includes instructions for participants to compare the line drawings to their own bare feet while standing and to select the picture that best represents their left and right feet in turn. Participants were asked about the presence of pain, aching or stiffness in the feet in the past month, categorised as occurring on no days, a few days, some days, most days or all days. The questionnaire also asked about consultation with their general practitioner and other health care professionals for foot problems within the preceding 12 months, and to indicate the location of foot pain experienced in the right and left feet in the past month by shading on a foot manikin (© The University of Manchester 2000. All rights reserved) [21]. Further validated questionnaires included the Manchester Foot Pain and Disability Index (MFPDI) [22], Generalized Anxiety Disorder Assessment (GAD7) [23], and Patient Health Questionnaire (PHQ9) [24]. Socioeconomic characteristics included marital status, higher education, current employment status, and ethnicity. The questionnaire also asked about self-reported height and weight, comorbidities (diabetes mellitus, hypertension, hyperlipidaemia, myocardial infarction, angina, stroke and transient ischaemic attack), and alcohol consumption.

Non-responders to the questionnaire were sent a reminder postcard after two weeks. Those who did not respond to the reminder postcard were sent a repeat questionnaire four weeks after the initial mailing. Participants were asked to consent to review of their medical records by the research team. The presence of tophi and serum uric acid (SUA) levels (highest level recorded) were ascertained from medical records of consenting participants over the preceding two years.

### Case definitions

Hallux valgus was dichotomised as present or absent for each foot by classifying the three most severe grades as present and the two least severe grades as absent [20]. Foot pain was defined as reporting any pain, aching or stiffness in the feet on most or all days in the past month [9]. Foot pain location for each foot was determined from the foot manikin by using a transparent overlay and classified as 1^st^ MTPJ, hallux, lesser toes, mid-foot, ankle, plantar heel or posterior heel, as described previously [25]. Disabling foot pain was defined as reporting foot pain in the last month (as defined above) and reporting at least one of the ten items of the MFPDI function construct as occurring on most or every day(s) [26].

### Statistical analysis

In order to assess selective non-participation, we compared the age, gender and neighbourhood deprivation levels of the mailed sample, responders and non-responders using simple descriptive statistics. Neighbourhood deprivation was determined from the rank of the Indices of Multiple Deprivation (IMD) derived from the individual’s postal code, and split into tertiles for analysis [27].

Demographic, lifestyle, psychological, co-morbid and gout characteristics of survey responders were described using simple descriptive statistics. Body mass index (BMI) was calculated from self-reported height and weight. Anxiety was assessed using the GAD7 and categorised as none (score 0 to 4), mild (5 to 9), moderate (10 to 14) or severe (15 to 21) [23]. Depression was assessed using the PHQ9 and categorised as none (score 0 to 4), mild (5 to 9), moderate (10 to 14), moderately severe (15 to 19) and severe (20 to 27) [24]. Gout duration was calculated from reported age at first diagnosis.

Prevalence estimates for hallux valgus (unilateral and bilateral), foot pain in the last month and disabling foot pain were calculated in (1) survey responders using simple descriptive statistics and (2) the mailed population using logistic regression applying inverse probability weights based on age, gender and decile of deprivation rank to account for any initial selective non-response to the survey questionnaire. In those reporting foot pain in the last month, the percentage reporting pain in the 1^st^ MTPJ, hallux, lesser toes, mid-foot, ankle, plantar heel or posterior heel on the foot manikin were calculated.

IMD is a measure of neighbourhood deprivation, measured at the level of postcode. Therefore individuals within the sample may have been more similar to others within the same tertile of IMD score. For this reason, it was tested, using a variance components model [28], whether people within tertiles of IMD score were more similar to each other in terms of their foot pain characteristics (i.e., presence of hallux valgus, foot pain, disabling foot pain) than those in other tertiles. If this grouping was significant, a multi-level logistic regression model would be used (grouping individuals within tertiles), if it was non-significant, a single-level logistic regression model would be used.

Multi- or single-level logistic regression models were then applied as appropriate to assess the association of age, gender, neighbourhood IMD (tertile), BMI (<25, 25.0–29.9, 30.0–34.9, ≥35 kg/m^2^), anxiety (none, mild, moderate, severe), depression (none, mild, moderate, moderately severe, severe), diabetes mellitus, hypertension, hyperlipidaemia, ischaemic heart disease, cerebrovascular disease, gout duration, currently experiencing gout attack, number of attacks in last 12 months (0, 1–2, ≥3), oligo/polyarticular attacks, and current allopurinol use with hallux valgus, foot pain and disabling foot pain. Results are presented as odds ratios (OR) with 95 % CIs, unadjusted and then adjusted for the full range of variables described above.

In order to explore the influence of acute attacks of gout on ascertainment of foot pain and disabling foot pain, sensitivity analyses were carried out to determine the association of demographic and gout-related characteristics with foot pain and disabling foot pain, excluding those who reported experiencing a gout attack at the time of completing the questionnaire.

The percentage of respondents who had consulted a GP, physiotherapist or podiatrist/chiropodist about a foot problem in the last 12 months was calculated.

Missing data were infrequent, varying from 4 to 7 % on current gout attack, duration of gout, and BMI to 12 % for depression. Missing values were imputed using multiple imputation technique and all data then re-analyzed. Findings did not differ significantly therefore complete case analyses only are presented.

All analyses were conducted in Stata version 12.1 (Stata Corporation, Texas, USA).

## Results

### Study population

**Fig. 1:**
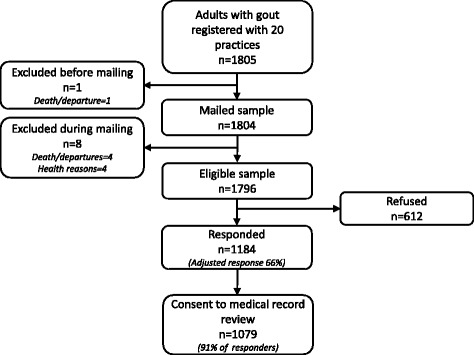
Flowchart showing participant recruitment

The baseline survey population consisted of 1805 people (Fig. 1), of whom nine were excluded during the mailing process (died, left the general practice or other serious health reasons). Of the remaining 1796 people, 1184 (66 %) responded to the baseline survey. Of these, 1079 (91 %) consented to their medical records being reviewed.

### Selective non-participation

Responders tended to be older than non-responders, with a higher proportion aged over 60 years (Table 1). They were also more likely to be male and live in less deprived areas.

**Table 1:** Age, gender and neighbourhood deprivation differences between mailed participants, non-responders and responders to the postal questionnaire. Numbers shown are n (%)

	All mailed participants	Non-responders and refusals	Responders
N	1805^a^	612	1184
Gender:			
Males	1471 (81.5)	476 (77.8)	990 (83.6)
Females	334 (18.5)	136 (22.2)	194 (16.4)
Age (years):			
Under 50	308 (17.1)	164 (26.8)	142 (12.0)
50–59	348 (19.3)	137 (22.4)	210 (17.7)
60–69	480 (26.6)	136 (22.2)	343 (29.0)
70–79	448 (24.8)	107 (17.5)	339 (28.6)
80 and over	221 (12.2)	68 (11.1)	150 (12.7)
Age (years), males:			
Under 50	294 (20.0)	155 (32.6)	138 (13.9)
50–59	301 (20.5)	113 (23.7)	187 (18.9)
60–69	401 (27.3)	108 (22.7)	293 (29.6)
70–79	340 (23.1)	65 (13.7)	273 (27.6)
80 and over	135 (9.2)	35 (7.4)	99 (10.0)
Age (years), females:			
Under 50	14 (4.2)	9 (6.6)	4 (2.1)
50–59	47 (14.1)	24 (17.7)	23 (11.9)
60–69	79 (23.7)	28 (20.6)	50 (25.8)
70–79	108 (32.4)	42 (30.9)	66 (34.0)
80 and over	86 (25.8)	33 (24.3)	51 (26.3)
Neighbourhood deprivation (tertiles):			
Most deprived	609 (33.8)	237 (38.8)	369 (31.2)
Mid-deprived	595 (33.0)	189 (30.9)	405 (34.2)
Least deprived	600 (33.3)	185 (30.3)	410 (34.6)

^a^Nine of these participants were excluded during the mailing process

### Characteristics of survey responders

Mean age of responders was 65.6 (SD 12.5) years. Responders were predominantly male (Table 1), married/cohabiting, white and had not attended higher education (Table 2). Just over half were retired. Over 80 % were overweight or obese (BMI > 25 kg/m^2^). Self-reported comorbidities were common, with hypertension most prevalent followed by hyperlipidaemia and then diabetes and vascular disease. Approximately one-quarter reported symptoms of anxiety and depression. Almost half drank alcohol at least three times per week with 113 (9.7 %) reporting never drinking.

**Table 2:** Demographic, lifestyle, psychological, co-morbid and gout characteristics of survey responders (*n* = 1184)

	n (%)
Marital status:	
Married/Co-habiting	882 (75.7)
Divorced/separated	91 (7.8)
Widowed	114 (9.8)
Single	78 (6.7)
Higher education:	
Yes	249 (22.3)
No	870 (77.8)
Employment status:	
Employed	401 (34.9)
Retired	656 (57.1)
Not working	35 (3.1)
Other^a^	56 (4.9)
Ethnicity:	
White	1126 (97.6)
Non-white	28 (2.4)
BMI (kg/m^2^):	
<25.0	221 (19.8)
25.0–29.9	511 (45.7)
30.0–34.9	260 (23.2)
≥35.0	127 (11.4)
Anxiety:	
None	844 (77.2)
Mild	141 (12.9)
Moderate	64 (5.9)
Severe	45 (4.1)
Depression:	
None	763 (73.2)
Mild	148 (14.2)
Moderate	65 (6.2)
Moderately severe	40 (3.8)
Severe	26 (2.5)
Co-morbid conditions:	
Diabetes mellitus	205 (17.3)
Hypertension	731 (61.7)
Hyperlipidaemia	508 (42.9)
Ischaemic heart disease	215 (18.2)
Cerebrovascular disease	86 (7.3)
Alcohol consumption:	
Daily/almost daily	273 (23.4)
3–4 times per week	263 (22.5)
1–2 times per week	254 (21.8)
1–3 times per month	109 (9.3)
Occasionally	155 (13.3)
Never	113 (9.7)
Gout characteristics:	
Duration, years (mean, (SD))	11.9 (12.1)
Current attack	132 (11.6)
Number of attacks in last 12 months:	
0	398 (35.4)
1–2	418 (37.2)
≥3	307 (27.3)
Oligo/polyarticular attacks	436 (38.6)
Current allopurinol use	630 (56.3)
Tophi	25 (2.3)^b^
Highest SUA (mean (SD), μmol/l)	441 (116)^b,c^

*BMI* body mass index, *SD* standard deviation, *SUA* serum uric acid

^a^Includes those who are off work sick for less than 6 months, in education, looking after the home/children

^b^In those with permission to access medical records (*n* = 1079)

^c^in 461 people with one or more measurement of uric acid recorded

Mean gout duration was 11.9 (SD 12.1) years. Having at least one attack of gout in the last 12 months was reported by 725 people (64.5 %) and 132 (11.6 %) reported currently having an attack of gout. Current allopurinol use was reported by 620 (56.3 %). Of the 1079 people who consented to medical record review, 25 (2.3 %) had the presence of tophi recorded in the last two years, whilst SUA level was recorded in the preceding two years in 461 (42.7 %) (mean 441 μmol/l, SD 116).

### Prevalence of hallux valgus, foot pain and disabling foot pain

Unilateral hallux valgus, bilateral hallux valgus, foot pain in the last month and disabling foot pain were reported by 203 (17.9 %), 215 (19.0 %), 248 (22.1 %) and 155 (14.4 %) responders respectively. On applying the sampling weights, these equate to estimated prevalences in the mailed sample of 17.7 % (95 % CI 15.6 %, 20.1 %) for unilateral hallux valgus, 18.6 % (95 % CI 16.4 %, 21.0 %) for bilateral hallux valgus, 22.3 % (95 % CI 19.9 %, 24.9 %) for foot pain in the last month, and 14.5 % (95 % CI 12.5 %, 16.8 %) for disabling foot pain.

Of those reporting foot pain in the last month, the 1^st^ MTPJ was the most common site (72.2 %), followed by the lesser toes (67.3 %), the midfoot (61.7 %), the hallux (58.9 %), ankle (54.0 %), posterior heel (35.1 %) and plantar heel (20.6 %).

### Factors associated with hallux valgus, foot pain, and disabling foot pain

The grouping of people into tertiles of IMD score accounted for <1.5 % of the overall amount of variation in hallux valgus, foot pain and disabling foot pain. Therefore, it was not necessary to take account of this grouping in analyses and single-level logistic regression models were used.

### Hallux valgus

In unadjusted analyses, hallux valgus was associated with age, female gender, mild and moderately- severe depression, and hypertension (Table 3). On multivariate analysis, only age (OR 1.47 per 10-year increase in age; 95 % CI 1.26, 1.72) and female gender (OR 2.03; 95 % CI 1.31, 3.15) remained associated with hallux valgus. There was no association with other sociodemographic factors, comorbidity, obesity, or gout characteristics.

**Table 3:** Factors associated with hallux valgus

	Hallux valgus n(%)	No hallux valgus n(%)	Unadjusted OR (95 % CI)	Adjusted OR^a^ (95 % CI)
N	418	714	-	-
Age, mean (SD)	68.7 (11.8)	63.6 (12.6)	1.41^b^ (1.27, 1.57)	1.47^b^ (1.26, 1.72)
Gender:				
Male	313 (74.9)	636 (89.1)	1.0	1.0
Female	105 (25.1)	78 (10.9)	2.74 (1.98, 3.78)	2.03 (1.31, 3.15)
Neighbourhood deprivation tertiles:				
Most deprived	131 (31.3)	219 (30.7)	1.0	1.0
Mid-deprived	148 (35.4)	236 (33.1)	1.05 (0.78, 1.41)	0.97 (0.67, 1.40)
Least deprived	139 (33.3)	259 (36.3)	0.90 (0.67, 1.21)	1.02 (0.70, 1.48)
BMI (kg/m^2^):				
<25.0	83 (21.5)	132 (19.2)	1.0	1.0
25.0–29.9	171 (44.2)	315 (45.9)	0.86 (0.62, 1.20)	1.04 (0.70, 1.56)
30.0–34.9	88 (22.7)	162 (23.6)	0.86 (0.59, 1.26)	1.29 (0.81, 2.05)
≥35.0	45 (11.6)	77 (11.2)	0.93 (0.59, 1.47)	1.21 (0.68, 2.16)
Anxiety:				
None	287 (74.0)	534 (79.4)	1	1
Mild	52 (13.4)	82 (12.2)	1.18 (0.81, 1.72)	1.04 (0.61, 1.77)
Moderate	29 (7.5)	32 (4.8)	1.69 (1.00 2.84)	1.37 (0.60, 3.10)
Severe	20 (5.2)	25 (3.7)	1.49 (0.81, 2.73)	0.83 (0.31, 2.25)
Depression:				
None	249 (68.0)	489 (76.4)	1	1
Mild	62 (16.9)	82 (12.8)	1.48 (1.03, 2.14)	1.36 (0.85, 2.19)
Moderate	23 (6.3)	38 (5.9)	1.19 (0.69, 2.04)	1.33 (0.62, 2.87)
Moderately severe	20 (5.5)	18 (2.8)	2.18 (1.13, 4.20)	1.39 (0.52, 3.72)
Severe	12 (3.3)	13 (2.0)	1.81 (0.82, 4.03)	1.83 (0.53, 6.37)
Comorbidity:				
Diabetes mellitus	73 (17.5)	119 (16.7)	1.06 (0.77, 1.46)	0.69 (0.46, 1.06)
Hypertension	279 (66.8)	420 (58.8)	1.41 (1.09, 1.81)	0.83 (0.58, 1.17)
Hyperlipidaemia	192 (45.9)	299 (41.9)	1.18 (0.92, 1.50)	1.18 (0.85, 1.62)
Ischaemic heart disease	86 (20.6)	121 (17.0)	1.26 (0.93, 1.73)	0.89 (0.59, 1.33)
Cerebrovascular disease	35 (8.4)	46 (6.4)	1.33 (0.84, 2.10)	1.14 (0.70, 2.18)
Gout characteristics:				
Duration, mean (SD)	12.7 (12.9)	11.4 (11.6)	1.09^c^ (0.98, 1.21)	1.14^c^ (0.99, 1.31)
Current attack	54 (13.6)	72 (10.4)	1.34 (0.93, 1.97)	1.07 (0.63, 1.82)
Number of attacks in last 12 months:				
0	136 (34.3)	244 (35.8)	1.0	1.0
1–2	152 (38.1)	251 (36.8)	1.08 (0.81, 1.44)	1.06 (0.73, 1.54)
≥3	109 (27.5)	187 (27.4)	1.05 (0.76, 1.43)	1.12 (0.72, 1.75)
Oligo/polyarticular attacks	159 (39.9)	261 (38.0)	1.08 (0.84, 1.39)	0.96 (0.69, 1.33)
Current allopurinol use	222 (56.5)	379 (55.7)	1.03 (0.80, 1.32)	1.06 (0.75, 1.49)

*BMI* body mass index, *CI* confidence interval, *OR* odds ratio, *SD* standard deviation

^a^OR adjusted for age, gender, deprivation, BMI, anxiety, depression, comorbidity and gout characteristics

^b^per 10-year increase in age

^c^per 10-year increase in duration of disease

### Foot pain

In unadjusted analyses, foot pain was associated with female gender, being obese/severely obese, anxiety, depression, diabetes mellitus, ischaemic heart disease, currently having an attack of gout, the number of gout attacks in the previous 12 months, and polyarticular attacks (Table 4). On multivariate analysis, foot pain remained associated with obesity (obese OR 2.23; 95 % CI 1.16, 4.27; severely obese OR 2.61; 95 % CI 1.25, 5.45), moderately-severe (OR 3.41; 95 % CI 1.22, 9.57) and severe depression (OR 4.55; 95 % CI 1.20, 17.18), currently experiencing a gout attack (OR 2.38; 95 % CI 1.35, 4.20), and oligo/polyarticular attacks (OR 1.67; 95 % CI 1.11, 2.51).

**Table 4:** Factors associated with foot pain in the last month

	Foot pain in last month n (%)	No foot pain in last month n (%)	Unadjusted OR (95 % CI)	Adjusted^a^ OR (95 % CI)
N	248	875	-	-
Age, mean (SD)	66.6 (11.7)	65.0 (12.7)	1.11^b^ (0.99, 1.24)	1.14^b^ (0.94, 1.38)
Gender:				
Male	190 (76.6)	752 (85.9)	1.0	1.0
Female	58 (23.4)	123 (14.1)	1.87 (1.31, 2.65)	1.33 (0.77, 2.30)
Neighbourhood deprivation tertiles:				
Most deprived	93 (37.5)	258 (29.5)	1.0	1.0
Mid-deprived	78 (31.5)	304 (34.7)	0.71 (0.50, 1.00)	0.81 (0.50, 1.31)
Least deprived	77 (31.1)	313 (35.8)	0.68 (0.48, 0.96)	1.14 (0.71, 1.82)
BMI (kg/m^2^):				
<25.0	34 (15.2)	177 (21.1)	1.0	1.0
25.0–29.9	90 (40.2)	397 (47.2)	1.18 (0.77, 1.82)	1.64 (0.89, 2.99)
30.0–34.9	62 (27.7)	182 (21.6)	1.77 (1.11, 2.83)	2.23 (1.16, 4.27)
≥35.0	38 (17.0)	85 (10.1)	2.33 (1.37, 3.95)	2.61 (1.25, 5.45)
Anxiety:				
None	133 (60.7)	677 (81.8)	1.0	1.0
Mild	41 (18.7)	96 (11.6)	2.17 (1.44, 3.28)	1.05 (0.57, 1.95)
Moderate	25 (11.4)	34 (4.1)	3.74 (2.16, 6.48)	1.38 (0.56, 3.39)
Severe	20 (9.1)	21 (2.5)	4.85 (2.56, 9.19)	1.21 (0.41, 3.51)
Depression:				
None	93 (47.0)	644 (80.2)	1.0	1.0
Mild	52 (26.3)	90 (11.2)	4.00 (2.67, 6.00)	3.02 (1.78, 5.11)
Moderate	20 (10.1)	43 (5.4)	3.22 (1.82, 5.71)	1.42 (0.58, 3.46)
Moderately severe	20 (10.1)	18 (2.2)	7.69 (3.93, 15.08)	3.41 (1.22, 9.57)
Severe	13 (6.6)	8 (1.0)	11.25 (4.54, 27.88)	4.55 (1.20, 17.18)
Comorbidity:				
Diabetes mellitus	59 (23.8)	134 (15.3)	1.73 (1.22, 2.44)	1.00 (0.60, 1.66)
Hypertension	166 (66.9)	529 (60.5)	1.32 (0.98, 1.78)	0.98 (0.62, 1.57)
Hyperlipidaemia	117 (47.2)	374 (42.7)	1.20 (0.90, 1.59)	1.12 (0.74, 1.71)
Ischaemic heart disease	56 (22.6)	140 (16.0)	1.53 (1.08, 2.17)	1.43 (0.87, 2.35)
Cerebrovascular disease	18 (7.3)	62 (7.1)	1.03 (0.60, 1.77)	0.83 (0.40, 1.69)
Gout characteristics:				
Duration, mean (SD)	12.7 (13.4)	11.8 (11.8)	1.06^c^ (0.94, 1.20)	1.05^c^ (0.88, 1.25)
Current attack	62 (26.1)	65 (7.7)	4.21 (2.87, 6.19)	2.38 (1.35, 4.20)
Number of attacks in last 12 months:				
0	63 (27.2)	313 (37.5)	1.0	1.0
1–2	77 (33.2)	323 (38.7)	1.18 (0.82, 1.71)	0.96 (0.58, 1.59)
≥3	92 (39.7)	199 (23.8)	2.30 (1.59, 3.31)	1.17 (0.66, 2.05)
Oligo/polyarticular attacks	132 (56.4)	289 (34.4)	2.47 (1.84, 3.32)	1.67 (1.11, 2.51)
Current allopurinol use	144 (62.1)	463 (55.6)	1.31 (0.97, 1.76)	1.11 (0.72, 1.72)

*BMI* body mass index, *CI* confidence interval, *OR* odds ratio, *SD* standard deviation

^a^OR adjusted for age, gender, deprivation, BMI, anxiety, depression, comorbidity and gout characteristics

^b^per 10-year increase in age

^c^per 10-year increase in duration of disease

Sensitivity analysis excluding those with a current attack of gout (*n* = 132) did not alter the findings: age (OR 1.24 per 10-year increase in age; 95 % CI 1.00, 1.55), obesity (obese OR 2.67; 95 % CI 1.32, 5.38: severely obese OR 3.16; 95 % CI 1.44, 6.93), mild depression (OR 2.04; 95 % CI 1.09, 3.81) and oligo/polyarticular attacks (OR 1.86; 95 % CI 1.18, 2.95) were significantly associated with the presence of foot pain in the last month.

### Disabling foot pain

Disabling foot pain was associated with female gender, obesity, anxiety, depression, diabetes mellitus, hyperlipidaemia, ischaemic heart disease, currently having a gout attack, the number of gout attacks in the previous 12 months, and oligo/polyarticular attacks, on univariate analysis (Table 5). In adjusted analyses, only obesity (obese OR 2.44; 95 % CI 1.10, 5.41; severely obese OR 3.10; 95 % CI 1.28, 7.47), depression (moderately severe OR 4.16; 95 % CI 1.28, 13.51; severe OR 8.81; 95 % CI 2.04, 38.01), ischaemic heart disease (OR 1.84; 95 % CI 1.06, 3.20) and currently having an attack of gout (OR 2.63; 95 % CI 1.39, 4.98) remained significant.

**Table 5:** Factors associated with disabling foot pain

	Disabling foot pain in last month n(%)	No disabling foot pain in last month^a^ n(%)	Unadjusted OR (95 % CI)	Adjusted^b^ OR (95 % CI)
N	155	923		
Age, mean (SD)	66.3 (11.9)	64.8 (12.6)	1.11^c^ (0.96, 1.27)	1.24^c^ (0.98, 1.56)
Gender:				
Male	120 (77.4)	796 (86.2)	1.0	1.0
Female	35 (22.6)	127 (13.8)	1.83 (1.20, 2.78)	1.59 (0.85, 2.95)
Neighbourhood deprivation tertiles:				
Most deprived	64 (41.3)	273 (29.6)	1.0	1.0
Mid-deprived	46 (29.7)	316 (34.2)	0.62 (0.41, 0.94)	0.83 (0.47, 1.46)
Least deprived	45 (29.0)	334 (36.2)	0.57 (0.38, 0.87)	0.96 (0.55, 1.68)
BMI (kg/m^2^):				
<25.0	19 (13.4)	185 (20.9)	1.0	1.0
25.0–29.9	54 (38.0)	418 (47.1)	1.26 (0.73, 2.18)	1.73 (0.82, 3.64)
30.0–34.9	41 (28.9)	195 (22.0)	2.05 (1.15, 3.66)	2.44 (1.10, 5.41)
≥35.0	28 (19.7)	89 (10.0)	3.06 (1.62, 5.78)	3.10 (1.28, 7.47)
Anxiety:				
None	83 (55.3)	714 (81.7)	1.0	1.0
Mild	31 (20.7)	101 (11.6)	2.64 (1.66, 4.19)	1.13 (0.56, 2.28)
Moderate	20 (13.3)	37 (4.2)	4.65 (2.58, 8.38)	1.53 (0.56, 4.20)
Severe	16 (10.7)	22 (2.5)	6.26 (3.16, 12.39)	1.05 (0.31, 3.55)
Depression:				
None	55 (39.6)	675 (79.8)	1.0	1.0
Mild	39 (28.1)	99 (11.7)	4.83 (3.05, 7.67)	3.27 (1.80, 5.97)
Moderate	16 (11.5)	45 (5.3)	4.36 (2.32, 8.22)	2.45 (0.92, 6.51)
Moderately severe	16 (11.5)	19 (2.3)	10.33 (5.03, 21.22)	4.16 (1.28, 13.51)
Severe	13 (9.4)	8 (1.0)	19.94 (7.93, 50.17)	8.81 (2.04, 38.01)
Comorbidity:				
Diabetes mellitus	41 (26.5)	141 (15.3)	1.99 (1.34, 2.97)	0.90 (0.50, 1.62)
Hypertension	106 (68.4)	561 (60.8)	1.40 (0.97, 2.01)	0.81 (0.46, 1.42)
Hyperlipidaemia	86 (55.5)	392 (42.5)	1.69 (1.20, 2.38)	1.38 (0.83, 2.29)
Ischaemic heart disease	45 (29.0)	145 (15.7)	2.19 (1.49, 3.24)	1.84 (1.06, 3.20)
Cerebrovascular disease	9 (5.8)	66 (7.2)	0.80 (0.39, 1.64)	0.67 (0.29, 1.57)
Gout characteristics:				
Duration (mean (SD))	13.5 (13.9)	11.7 (11.7)	1.12^d^ (0.98, 1.29)	1.17^d^ (0.96, 1.42)
Current attack	48 (32.2)	72 (8.1)	5.39 (3.54, 8.19)	2.63 (1.39, 4.98)
Number of attacks in last 12 months:				
0	39 (26.4)	328 (37.3)	1.00	1.00
1–2	44 (29.7)	341 (38.8)	1.09 (0.69, 1.71)	0.94 (0.51, 1.73)
≥3	65 (43.9)	211 (24.0)	2.59 (1.59, 3.99)	1.26 (0.64, 2.47)
Oligo/polyarticular attacks	87 (58.8)	310 (35.0)	2.65 (1.86, 3.78)	1.59 (0.98, 2.58)
Current allopurinol use	91 (62.3)	490 (55.8)	1.31 (0.91, 1.88)	1.13 (0.67, 1.90)

*BMI* body mass index, *CI* confidence interval, *OR* odds ratio, *SD* standard deviation

^a^no foot pain or foot pain that is not disabling

^b^OR adjusted for age, gender, deprivation, BMI, anxiety, depression, comorbidity and gout characteristics

^c^per 10-year increase in age

^d^per 10-year increase in duration of disease

After exclusion of those people reporting a current attack of gout, age (OR 1.42 per 10-year increase in age; 1.08, 1.87), obesity (obese OR 3.73; 95 % CI 1.54, 9.09: severely obese OR 4.36; 95 % CI 1.64, 11.64), depression (mild OR 2.63; 95 % CI 1.25, 5.53; moderate OR 3.53; 95 % CI 1.11, 11.26) and ischaemic heart disease (OR 2.45; 95 % CI 1.32, 4.53) were significantly associated with disabling foot pain in the last month.

### Utilisation of health services

495 respondents (42.8 %) reported consulting their GP in the previous 12 months about their feet, whilst 72 (6.1 %) reported seeing a physiotherapist and 281 (23.7 %) consulting a podiatrist/chiropodist.

## Discussion

In this large primary-care based study of people with gout, foot problems were prevalent. Over one-third had unilateral or bilateral hallux valgus and one-fifth had experienced pain, aching or stiffness in the foot on most days in the preceding month. Amongst those with foot pain almost two-thirds reported disabling symptoms. Hallux valgus was associated with age and female gender but no association was seen with gout-related or comorbid features. Foot pain and related disability both associated with age, obesity and depression, however foot pain was associated with a history of polyarticular gout attacks whereas disabling foot pain was associated with ischaemic heart disease. Foot problems were a common reason to consult a health professional, with almost one-half seeing their GP in the last 12 months and nearly one-quarter a podiatrist/chiropodist.

There are few previous studies of foot problems in people with gout in primary care. A small primary-care based study found a similar prevalence of hallux valgus of 41 % [4]. The prevalence of chronic big toe pain was 16 % which is consistent with our finding that of the 22 % who reported foot pain in the preceding month, the 1^st^ MTPJ (72 %) and hallux (59 %) were commonly affected. It is noteworthy, however, that the lesser toes, mid-foot, ankle, plantar heel and posterior heel were also commonly painful. In this previous study [4], both hallux valgus and big toe pain were more than twice as common in people with gout as matched gout-free control subjects. Our current findings did not find evidence to support a role for gout-specific or comorbid factors in the increased prevalence of hallux valgus in gout. There are various risk factors described for hallux valgus which were not assessed in our study, for example footwear, foot posture and 1^st^ MTPJ OA [6–8]. Similarly foot pain in the general population has been shown to associate with obesity and footwear [9–11]. Whilst footwear worn by people with gout is frequently suboptimal [16] and gout occurs more frequently at osteoarthritic 1^st^ MTPJs [29, 30], little is known about foot posture in gout. The specific association observed between disabling foot pain and ischaemic heart disease is an interesting finding. The explanation for this observation is not clear but it is possible that disabling foot pain arises from other conditions associated with ischaemic heart disease that we did not assess (for example, peripheral vascular disease) or is a marker of general frailty.

The frequency of consultation with a healthcare professional for foot problems was higher in people with gout in this study than that previously observed in people with foot problems in the general population (not specific to gout). In our study, 43 % of people with gout consulted their GP about foot problems in the preceding 12 months compared with 12 % of people with foot problems over 18 months in the general population [17]. Similarly, 24 % of people with gout consulted a podiatrist over 12 months compared to 18 % in the general population [18]. It is to be expected that the consultation prevalence should be lower for podiatrists than GPs, since consultation with a podiatrist in the UK generally requires a referral from a GP. These findings suggest that foot problems in people with gout may be more severe than in the general population emphasising the need to provide specific assessment and treatment of foot problems in people with gout.

This is the largest study of foot problems in people with gout to date. Further strengths include the primary care setting, ensuring relevance to the majority of patients with gout who are managed entirely in primary care, and the use of weighted logistic regression to account for non-response. Limitations of our study include the reliance on a primary care diagnosis of gout [31] although our previous work has shown this to be reasonably accurate [32, 33]. Response to the postal questionnaire was acceptable although responders were older, more likely to be male and were less socioeconomically deprived. This was partially addressed by inverse probability weighting based on these variables. The external validity of our findings is reduced by the low representation of ethnic minority groups which is typical of the source population [34]. The study assessed hallux valgus using a validated line-drawing instrument [20] which was included in the postal questionnaire. Ascertainment of hallux valgus via self-report, rather than clinical assessment, risks under-estimating its prevalence [35]. Other 1^st^ MTPJ deformities such as hallux rigidus might also be relevant to people with gout but could not be assessed in a postal self-report questionnaire. A further limitation is that the underlying cause of foot pain is not known but possible causes include attacks of acute gout, chronic erosive arthropathy, co-existent OA, pain related to comorbidity (for example peripheral vascular disease), or soft tissue pathology. The possibility that foot pain in gout could relate to comorbid conditions or arise from soft tissue structures is supported by our findings that disabling foot pain was associated with ischaemic heart disease and that pain in the plantar heel and posterior heel was prevalent.

Our findings suggest that patients with gout should undergo comprehensive clinical assessment of their feet and be offered specific multidisciplinary treatment for foot problems, such as gout-specific foot education and promotion, foot orthoses and good footwear [36, 37]. The association of foot pain with gout characteristics (history of oligo/polyarticular attacks) provides justification for ensuring optimal use of urate-lowering therapy to achieve target serum urate levels. Further research is needed to ascertain the causes of disabling chronic foot pain in gout, via clinical assessment, and to develop evidence-based treatments for foot problems in people with gout.

## Conclusions

Foot problems are common findings in people with gout and frequently lead to consultation with healthcare professionals. One-third of people with gout have hallux valgus and approximately one-quarter report experiencing foot pain in the last month. Nearly two-thirds of those with foot pain report disabling symptoms. Hallux valgus associated with age and gender, similar to the general population, whereas foot pain associates with obesity and gout characteristics, and disabling foot pain with obesity and comorbidity. These findings suggest that foot problems present significant difficulties to people with gout who need evidence-based, multidisciplinary foot-specific interventions.
